# Transpetrosal Approach to a Ruptured Distal Basilar Perforating Artery Aneurysm

**DOI:** 10.7759/cureus.34273

**Published:** 2023-01-27

**Authors:** Yasaman Alam, Travis Atchley, Nicholas Laskay, Andrew T Hale, Winfield S Fisher

**Affiliations:** 1 Neurological Surgery, University of Alabama at Birmingham, Birmingham, USA

**Keywords:** operative video, transpetrosal approach, cerebrovascular surgery, basilar perforating aneurysm, basilar artery aneurysm

## Abstract

Basilar perforating artery aneurysms are rare and underreported vascular anomalies in the cerebrovascular literature. Various open and endovascular treatment approaches can be employed to treat these aneurysms based on several patient- and aneurysm-specific factors. Some authors have even advocated for conservative, nonoperative management. Here, we report a case of a ruptured distal basilar perforating artery aneurysm secured by an open transpetrosal approach.

A 67-year-old male presented to our institution with a Hunt-Hess grade 2, modified Fisher grade 3 subarachnoid hemorrhage (SAH). Initial cerebral digital subtraction angiography (DSA) did not identify an intracranial aneurysm or other vascular lesions. However, the patient had a re-rupture event several days after presentation. DSA at this time revealed a posteriorly projecting distal basilar perforating artery aneurysm. Initial attempts with endovascular coil embolization were unsuccessful. Thus, an open transpetrosal approach was taken to gain access to the middle and distal basilar trunk to secure the aneurysm.

This case underscores the unpredictability of basilar perforating artery aneurysms and the challenges encountered when considering active treatment. We demonstrate an open surgical approach with an intraoperative video for definitive management after failed attempted endovascular treatment.

## Introduction

Basilar perforator aneurysms can arise directly from the basilar trunk or from the basilar perforating arteries. These aneurysms are rare and are underreported in the neurovascular literature. They were first reported by Ghogawala et al. (1996), and there have been less than 50 cases reported since [1]. Satti et al. (2016) proposed a three-type classification system for these aneurysms based on their anatomical location and configuration [2]. Because these aneurysms are rare, the natural history and optimal treatment modality are not firmly established. Furthermore, these aneurysms can often be angiographically occult on initial imaging, only to be discovered on routine follow-up angiography or at re-rupture [3-9]. If a basilar perforating artery aneurysm is found, then various treatment and management options are available [1,10-15]. Some authors have advocated conservative management and close follow-up [4,6,7,16]. In a systematic review by Granja et al. (2020), there was no observed difference in the primary outcome between conservative and treatment groups [17]. Nevertheless, aneurysmal re-rupture remains a concern and can place the patient at risk for significant morbidity and even mortality if efforts are not made to secure the aneurysm. Here, we present an open surgical approach to secure a basilar perforator aneurysm, initially angiographically occult, only later to be discovered after re-rupture.

## Case presentation

A 67-year-old male with diabetes mellitus, hypertension, and myasthenia gravis, on aspirin 81 mg, presented with a Hunt-Hess grade 2, modified Fisher grade 3 subarachnoid hemorrhage (SAH).

**Figure 1:**
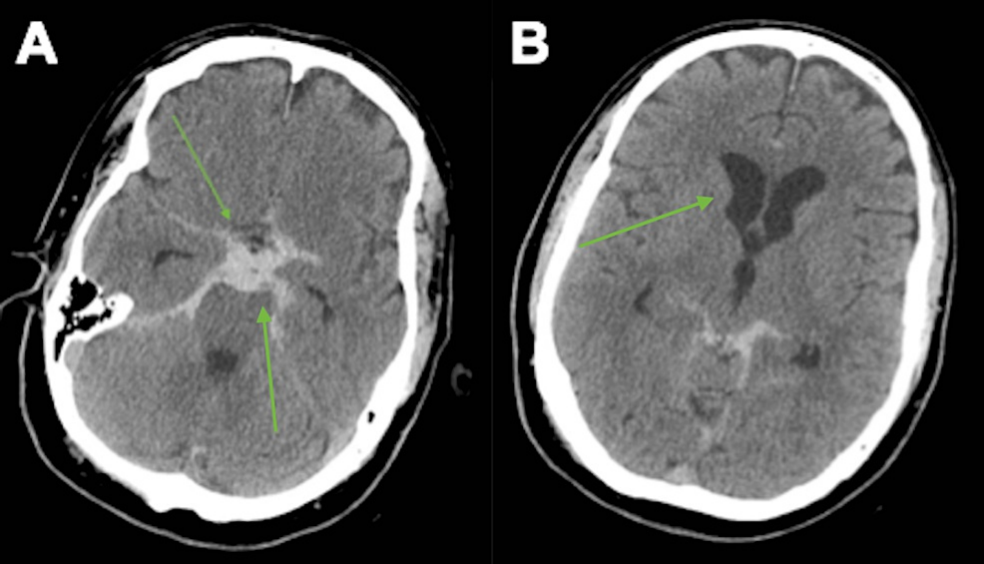
Axial computed tomography of the head demonstrating extensive diffuse subarachnoid hemorrhage involving the premedullary and prepontine cisterns extending into the basal cisterns (A) and evidence of early obstructive hydrocephalus (B)

**Figure 2:**
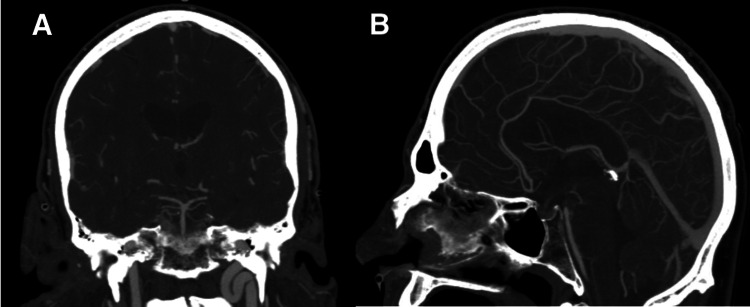
Computed tomography angiography of the head (mean intensity projection) without evidence of basilar perforator aneurysm in the sagittal (A) nor the coronal (B) plane

On physical examination, the patient was hemodynamically stable and neurologically intact. Non-contrast computed tomography (CT) of the head revealed diffuse SAH and associated ventriculomegaly (Figure 1). Initial CT angiography (CTA) as well as cerebral digital subtraction angiography (DSA) did not reveal an aneurysm or other vascular lesion (Figure 2). Nevertheless, the patient was admitted to the neuro-intensive care unit (ICU) for close observation.

The patient’s hospital course was initially uncomplicated. He was participating in physical therapy when he acutely became unresponsive, requiring intubation for airway protection. CTA at this time demonstrated worsening, diffuse SAH with progressive ventriculomegaly. Additionally, a posteriorly projecting distal basilar perforating artery aneurysm measuring approximately 2 × 1.5 × 1 mm was now identifiable. The family consented to all subsequent procedures, and an emergent external ventricular drain was placed.

**Figure 3:**
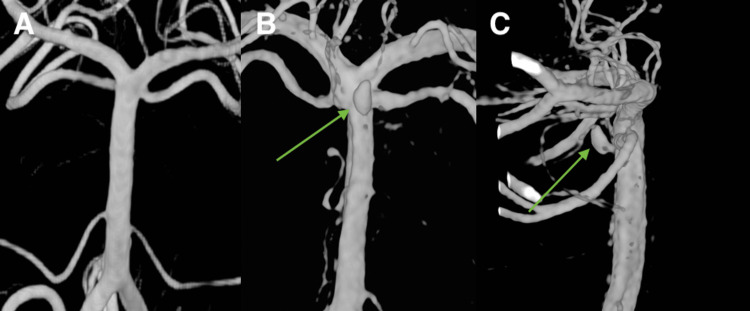
Three-dimensional reconstructions from digital subtraction angiography post-bleed day 1 demonstrating angiographic occult perforator aneurysm arising from the distal basilar artery near its junction with the right superior cerebellar artery (A) and a similar angled reconstruction created during digital subtraction angiography following re-rupture (B) as well as lateral view of the aneurysm (C)

He was taken to the angiography suite where a small non-trunk basilar perforator aneurysm was identified (Figure 3). Opacification of this aneurysm during balloon occlusion of the right superior cerebellar artery (SCA) confirms its origin from the basilar artery itself rather than the SCA. The aneurysm was overall slow to opacify, and its neck was not well visualized.

The operators performed two separate attempts at endovascular treatment of the aneurysm, both of which were unsuccessful due to inability to advance microcatheters and guidewires for aneurysm selection.

Due to the patient’s extensive blood burden and concern that dual antiplatelet therapy may further destabilize any residual aneurysmal thrombus, pipeline flow diversion treatment is deemed too unsafe for the patient. After careful consideration, we decided to attempt clipping the aneurysm with an open, transpetrosal approach (Video 1). This surgery proceeded without incident, and the patient returned to the neuro-ICU following the case.

**Video 1:** Operative approach to a ruptured distal basilar perforating artery aneurysm

**Figure 4:**
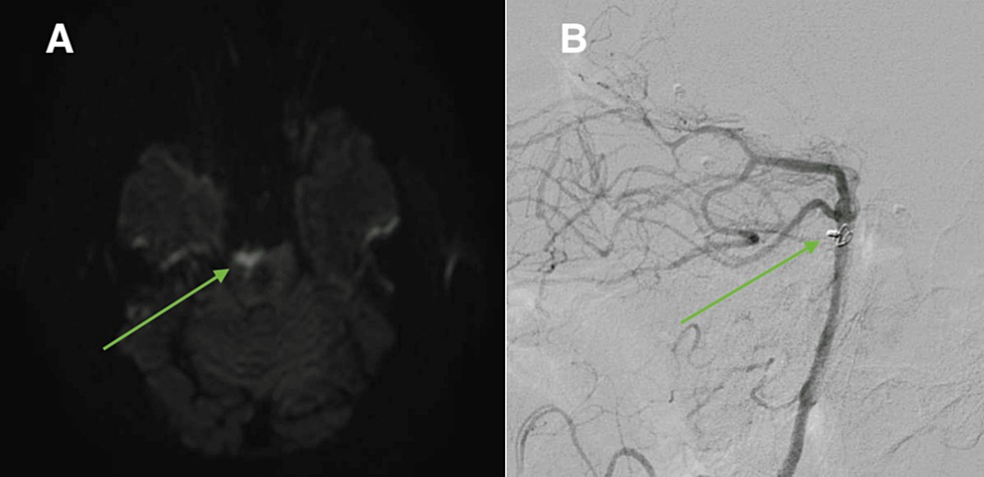
Small area of diffusion-weighted restriction in the right anterior portion of the rostral pons (A); right anterior oblique view of the basilar artery during postoperative digital subtraction angiography showing complete aneurysmal obliteration (B)

Magnetic resonance imaging was obtained after surgery, which demonstrated a small area of diffusion-weighted restriction in the right anterior portion of the rostral pons, suggestive of infarct from occluding the perforator (Figure 4A). Postoperative cerebral DSA confirmed obliteration of aneurysm from circulation (Figure 4B).

The patient ultimately required a ventriculoperitoneal shunt, a tracheostomy, and a percutaneous gastrostomy tube. He had right-sided oculomotor and trochlear nerve palsies with left hemiparesis. He was transferred to a special care unit for ventilator weaning and was ultimately discharged home off of the ventilator. At the last follow-up in March 2022, he was ambulatory with markedly improved left-sided strength, and he is independent with most activities.

## Discussion

Ruptured basilar perforating artery aneurysms are uncommon causes of SAH. While there remains a growing body of literature characterizing these entities, these aneurysms remain exceedingly rare and consequently difficult to predict. We aim to add to this growing body by presenting a case of a transpetrosal approach to clip a posteriorly projecting ruptured basilar perforator aneurysm.

Recently, basilar perforating artery aneurysms were classified into three types by Satti et al. (2017) (Table 1) [2]. Here, we present a case of a type IIb posteriorly projecting perforating artery aneurysm at the level of the distal basilar artery, adjacent to the right SCA takeoff. Several other studies have demonstrated similar locations, often a difficult area to expose using open approaches [1,12].

**Table 1:** Classification system for basilar artery perforator aneurysm

Type of aneurysm	Location of aneurysm
Type 1	Aneurysm arises directly from the trunk of the basilar artery, not involving the perforator
Type IIa	Aneurysm incorporates the origin of the perforator
Type IIb	Perforating artery arises from the dome of the aneurysm
Type III	Aneurysm fusiform located along the distal portion of the perforating artery

After a review of the literature, only 50% of basilar perforator aneurysms are diagnosed at initial DSA [17]. This is likely attributable to acute thrombosis of the aneurysm after rupture. The incidence of thrombosis of small ruptured aneurysms has been shown to be 1%-2% [18]. In general, extremely small caliber aneurysms, such as basilar perforator aneurysms, are often characterized by reduced flow, facilitating partial or complete thrombosis [16,18,19]. This underscores the importance of close in-hospital observation of these patients and the utility of short-interval repeat DSA, especially if there is a considerable blood burden in the subarachnoid space. Considering this observation, the question remains: should active treatment of these aneurysms be considered at all?

In a systematic review by Granja et al. (2017), they concluded that in patients with non-trunk basilar perforating artery aneurysms (types II and III of the Satti classification), conservative expectant management may result in acceptable functional outcomes and low morbidity in patients not amenable to active treatment [2,17]. However, the natural history of basilar artery perforator aneurysms is not known. Thus, we caution against a purely conservative approach and that active treatment should be taken into consideration on an individualized basis. Our report provides anecdotal evidence to this point.

Approximately 70% of patients with non-trunk basilar perforating artery aneurysms found in the literature were treated actively, namely, by endovascular or open surgical means [17]. The majority were treated with a variety of endovascular techniques. However, the small caliber and narrow necks of these aneurysms provide a considerable technical challenge to the neurointerventionalist. In our case, coil embolization was unsuccessfully attempted twice. The concern for aneurysm instability remained high, so an open surgical approach was taken to secure the aneurysm. Despite morbidity in the short-term postoperative setting, the patient has improved dramatically, and there is no evidence of aneurysm recurrence at the last follow-up.

Of the cases reported in the literature, only six were managed with open intervention, often only after failed attempts at endovascular treatment [1,10,12,20]. At our institution, we prefer the transpetrosal approach for access to the middle and distal thirds of the basilar trunk for proximal and distal artery control and an unobstructed view of the aneurysm.

## Conclusions

Here, we present a case of an initially angiographically occult basilar perforating aneurysm with subsequent re-rupture. This case underscores the unpredictability of basilar perforating artery aneurysms and the challenges encountered when considering active treatment. The majority of these aneurysms are treated endovascularly in the modern era. However, it is important to recognize the relevance and indications for traditional open surgical intervention. We demonstrate a transpetrosal surgical approach to the basilar artery with an intraoperative video for definitive management after failed attempted endovascular treatment.
